# Correction: Whole-genome sequencing of chronic lymphocytic leukaemia reveals distinct differences in the mutational landscape between IgHV^mut^ and IgHV^unmut^ subgroups

**DOI:** 10.1038/s41375-019-0515-8

**Published:** 2019-07-17

**Authors:** A Burns, R Alsolami, J Becq, B Stamatopoulos, A Timbs, D Bruce, P Robbe, D Vavoulis, R Clifford, M Cabes, H Dreau, J Taylor, S J L Knight, R Mansson, D Bentley, R Beekman, J I Martín-Subero, E Campo, R S Houlston, K E Ridout, A Schuh

**Affiliations:** 1 Department of Molecular Haematology, Oxford Molecular Diagnostics Centre, John Radcliffe Hospital, Oxford, UK; 2 Department of Oncology, University of Oxford, Oxford, UK; 3 Nuffield Department of Clinical Laboratory Sciences, University of Oxford, Oxford, UK; 4 King Abdulaziz University, Faculty of Applied Medical Sciences, Jeddah, Saudi Arabia; 5 Illumina Cambridge Ltd, Saffron Walden, UK; 6 Department of Haematology, Oxford University Hospitals NHS Trust, Oxford, UK; 7 Wellcome Trust Centre for Human Genetics, University of Oxford, Oxford, UK; 8 Center for Hematology and Regenerative Medicine Huddinge, Karolinska Institute, Stockholm, Sweden; 9 Biomedical Epigenomics Group, Institut d’Investigacions Biomédiques August Pi i Sunyer (IDIBAPS), University of Barcelona, Barcelona, Spain; 10 Hematopathology Unit, Hospital Clinic, Institut d’Investigacions Biomèdiques August Pi i Sunyer (IDIBAPS), University of Barcelona, Barcelona, Spain; 11 Division of Molecular Pathology, The Institute of Cancer Research, London, UK

**Correction to: Leukemia**

10.1038/leu.2017.177

Published online 6 June 2017

This article was originally published under CC BY-NC-ND 4.0, but has now been made available under a CC BY 4.0 license. The PDF and HTML versions of the paper have been modified accordingly.

